# Spatial immune archetypes in gastric and colorectal cancer: a proposed conceptual framework for immunotherapy resistance and therapeutic remodeling

**DOI:** 10.3389/fimmu.2026.1859228

**Published:** 2026-06-11

**Authors:** Songlin Sun, Yicong Zeng, Jiahan Chen, Yang Zhong, Tong Zhou

**Affiliations:** Second Department of Gastrointestinal Surgery, Hepatobiliary, Pancreatic and Intestinal Diseases Research Institute, The Affiliated Hospital of North Sichuan Medical College, National Clinical Key Specialty (General Surgery), Sub-center of National Clinical Research Center for Digestive Diseases, Sichuan Clinical Research Center for Digestive Diseases, Nanchong, Sichuan, China

**Keywords:** colorectal cancer, gastric cancer, immunotherapy resistance, MSS/pMMR, spatial immune archetypes, spatial transcriptomics, translational medicine, tumor microenvironment

## Abstract

Gastric and colorectal cancers are leading causes of global cancer mortality, yet the transformative benefits of immune checkpoint blockade remain largely confined to the small fraction of patients with MSI-H/dMMR tumors. The vast majority—those with MSS/pMMR disease—exhibit primary resistance, underscoring an urgent need to look beyond bulk immune infiltration and dissect the *in situ* spatial determinants of immune evasion. We propose that immunotherapy outcome in these malignancies is dictated not by lymphocyte abundance alone, but by highly coordinated multicellular architectures—termed spatial immune archetypes—that govern immune exclusion, metabolic suppression, local activation, and epithelial-immune contact. Enabled by spatial transcriptomics, multiplexed proteomics, and multimodal computational integration, this review proposes a conceptual framework of four spatial archetypes synthesized from existing evidence across gastric and colorectal cancers: (i) the stromal-excluded barrier niche, characterized by CAF-mediated physical sequestration; (ii) the myeloid-suppressive metabolic niche, driven by TREM2+/SPP1TREM2+ macrophages and microbial crosstalk; (iii) the inflamed lymphoid-reactive niche, marked by mature tertiary lymphoid structures; and (iv) the epithelial-immune interface niche, a fragile surveillance equilibrium lost during immunoediting. We further examine how tissue-specific anatomical constraints and distinct microbial ecologies (*H. pylori* in the stomach, *F. nucleatum* in the colon) shape the prevalence of each archetype. By elucidating the molecular circuits and environmental dependencies underlying these spatially encoded resistance programs, we articulate a translational imperative: archetype-guided spatial biomarkers and targeted microenvironment-remodeling strategies provide the most viable framework to extend durable immunotherapeutic benefit to the historically refractory MSS/pMMR gastrointestinal cancer population.

## 1 Introduction

### 1.1 Unmet need in MSS/pMMR GI cancers

Gastric and colorectal malignancies now dominate global cancer incidence. Aging demographics and dietary transitions drive this epidemiology. Multimodal regimens—surgery, neoadjuvant chemoradiation, targeted systemic agents—deliver only incremental survival margins. Metastatic disease largely evades these standards (1–4). PD-1/PD-L1 blockade has altered clinical expectations for a discrete molecular subset. Objective tumor regression tracks with checkpoint inhibition. Current evidence indicates these gains concentrate almost exclusively within MSI-H/dMMR populations. Pan-tumor screens report 10–15% frequencies across unselected GI primaries (5, 6). Stage IV pathology diverges sharply from that baseline. Intense local immune surveillance eliminates highly immunogenic clones before vascular dissemination occurs. Immunoediting reduces metastatic prevalence to 4–7%. Clinical response rates mirror this biological filter. Single-agent ICI activity remains restricted to the MSI-H/dMMR minority. Primary resistance defines the remaining 93–96% of advanced MSS/pMMR cases (7–9). Accumulating clinical and translational evidence suggests that contemporary therapeutic bottlenecks stem less from compound availability and more from intrinsic tissue architecture. The tumor microenvironment (TME) operates as an active exclusion zone. Rather than serving as inert matrix, it establishes metabolic and stromal gradients that dismantle local immune surveillance. These mechanisms trigger early therapeutic failure. They operate upstream of classical adaptive evasion pathways (2, 10, 11).

### 1.2 Limitations of bulk and single-cell profiling

Histological and molecular profiling of GI malignancies still depend on reductionist frameworks. Standard practice combines bulk transcriptomics, tumor mutational burden (TMB) scoring, PD-L1 immunohistochemistry, and flow cytometric TIL enumeration. These assays establish baseline biomarkers. They fail to capture tissue architecture. Bulk RNA sequencing calculates population averages. This mathematical smoothing obscures low-abundance immunosuppressive niches. Single-cell RNA sequencing resolves transcriptional heterogeneity. Dissociation protocols strip cells from their native coordinates. Intercellular contact networks disintegrate during library preparation. The resulting dataset isolates cellular phenotypes from their microenvironmental ecology (12–14).

CD8+ T cell distribution illustrates this technical gap. Density measurements alone do not predict cytolytic output. Functional capacity hinges on three variables: physical proximity to malignant cells, micro-compartmentalization, and stromal permeability (15–17). Lymphocytes trapped within desmoplastic peritumoral bands remain metabolically inert. Baseline activation status and clonal expansion data lose relevance when physical barriers block antigen engagement. Standard dissociated workflows cannot distinguish true parenchymal infiltration from stromal entrapment (11, 18, 19). Current evidence indicates frequent discordance between bulk immune signatures and actual effector function. This spatial blind spot directly compromises prognostic stratification, as demonstrated by consistent discordance between bulk immune signatures and functional cytolytic output across GI cancer cohorts. It also limits our ability to forecast checkpoint inhibitor responsiveness in advanced MSS/pMMR cohorts (7, 20).

### 1.3 Spatial biology as a new analytical paradigm

Spatial biology now treats tissue architecture as a primary analytical variable. High-dimensional profiling platforms preserve transcriptomic and proteomic coordinates *in situ*. This methodology replaces isolated cellular phenotyping with neighborhood-level mapping. Cells do not function autonomously. They assemble into recurrent multicellular units—cellular neighborhoods, hubs, or niches (18, 21, 22). Current evidence indicates these structures operate as discrete signaling compartments. Localized paracrine signaling gradients, metabolic competition, and direct membrane contact govern collective behavior. Bulk averaging obscures compartmentalized dynamics. Spatial resolution restores the native tissue ecology (23–25). The investigative focus has shifted accordingly. Cataloging immune and malignant populations yields incomplete data. We now map structural organization and spatial restriction. It remains clear that physical proximity dictates functional output more reliably than phenotypic enumeration. Dysplastic crypts emit immunosuppressive signals that decay across micrometer distances. Early malignant transformation and subsequent immune evasion track with this spatial topography (16, 18, 21). Isolated genetic driver mutations initiate oncogenesis, but microenvironmental geometry sustains immune exclusion.

### 1.4 Spatial immune archetypes: conceptual framework and review scope

We define spatial immune archetypes as structurally conserved TME configurations that recur across independent patient cohorts. These architectures represent stable multicellular ecological equilibria. They differ from transient transcriptional programs or short-lived cellular states. Archetypal organization directly modulates disease progression kinetics and specific therapeutic vulnerabilities. This review concentrates on gastric and colorectal cancers. Both malignancies share endodermal and mucosal origins, recruiting analogous stromal and immune cellular building blocks. Anatomical constraints and mucosal architecture separate them: baseline luminal pH, epithelial geometry, and resident microbial ecologies diverge sharply. These environmental variables impose distinct selective pressures on local immune networks. We first evaluate high-plex spatial technologies that enable these measurements. We then delineate the structural components of tissue architecture and propose a conceptual taxonomy of spatial immune archetypes in GI malignancies. Each configuration carries distinct mechanistic implications. We hypothesize that this archetype-based model provides a framework for rational patient stratification and future clinical trial design. The objective remains direct: replace empirical biomarker screening with topology-driven treatment strategies and integrate these models into prospective clinical trial design.

## 2 Technologies and analytical framework

### 2.1 Single-cell multi-omics as molecular reference atlases

Spatial transcriptomics demands a precise molecular baseline. High-throughput scRNA-seq and scATAC-seq have systematically mapped transcriptional output and chromatin accessibility across the gastrointestinal TME. These dissociated profiles capture cellular heterogeneity. They define discrete states at single-cell resolution. Reference atlases provide the necessary vocabulary for spatial annotation—identifying SPP1+ macrophages in hypoxic niches, FAP+ fibroblasts remodeling the extracellular matrix, and CXCL13+ exhausted CD8+ T cells (14, 26–28). Dissociation, however, severs spatial relationships. The molecular signature survives, but the architectural map vanishes. Current evidence indicates that effector function in GI malignancies depends on immediate microenvironmental signals (12, 29, 30). A CD8+ T cell may carry identical exhaustion signatures in dissociated data, yet the upstream driver shifts with physical location. Precise coordinates separate distinct phenotypes: direct contact with regulatory T cells, entrapment in hypoxic cores, or structural blockage by dense stromal networks. We submit that single-cell multi-omics functions primarily as a reference dictionary rather than a standalone solution. When aligned with intact tissue sections, it translates isolated transcriptomic labels into spatial logic. This integration bridges the gap between dissociated sequencing and *in situ* tumor biology (13, 31, 32).

### 2.2 Spatial transcriptomic platforms

Spatial transcriptomics connects molecular phenotyping to tissue morphology. Current methodologies fall into two technical strategies. Array-based barcoding platforms—exemplified by 10x Genomics Visium—prioritize transcriptome-wide coverage over cellular resolution. Capture spots integrate signals from multiple cells (12, 33, 34). This limitation, however, becomes an asset for mapping tissue-scale architecture. These platforms reliably delineate macroscopic zones: immune-excluded stromal rims, hypervascularized angiogenic margins, inflamed lymphoid hotspots, necrotic tumor cores. For hypothesis-generating studies across large tissue sections, this breadth outweighs the loss of single-cell detail (12, 29, 33). High-resolution techniques operate differently. MERFISH and Slide-seq sacrifice whole-transcriptome scope to achieve single-cell or subcellular precision. The trade-off is deliberate. When the research question centers on cellular crosstalk, physical apposition matters more than comprehensive gene coverage (12, 14, 35). These methods resolve direct interfaces—malignant epithelial cells contacting specialized intraepithelial lymphocytes, for instance—at the frontline of immune surveillance. Current evidence indicates that such spatially resolved interactions often drive functional outcomes that bulk or dissociated data cannot capture. We argue that platform selection should follow the biological question, not technical novelty. Array-based maps guide regional annotation; high-resolution assays dissect cellular neighborhoods. Integrating both, when feasible, provides the most complete spatial inference (14, 25, 33).

### 2.3 Spatial proteomics and histology-linked validation

Spatial transcriptomics can generate compelling hypotheses—about signaling pathways or cellular states, for example. But proteins are the TME’s primary functional effectors. To capture this critical layer, highly multiplexed spatial proteomic tools now exist: multiplexed immunofluorescence (mIF), imaging mass cytometry (IMC), multiplexed ion beam imaging (MIBI), and co-detection by indexing (CODEX). They allow simultaneous visualization and quantification of dozens of protein markers on single, intact FFPE tissue sections (15, 30). These platforms empirically validate transcriptomic inferences. That is their indispensable role. They confirm complex protein co-expression patterns—for instance, verifying the phenotypic presence of ARG1+ and TREM2+ regulatory myeloid populations. At the same time, they capture dynamic post-translational modifications, including STAT3 or SMAD phosphorylation (7, 36, 37). Current evidence also indicates that spatial proteomics can physically substantiate putative ligand-receptor interactions initially predicted by algorithms such as NicheNet or CellChat (32, 38). Take CODEX analyses in colorectal cancer: they have helped delineate highly coordinated cellular neighborhoods at the invasive front. Such data provide quantitative evidence—effective antitumoral immunity depends on localized cellular clustering, not merely global immune cell abundance (7, 30).

### 2.4 Multimodal integration and archetype scoring

Spatial multi-omics data are high-dimensional. That complexity demands integration—multimodal, computational integration. Current analytical frameworks deploy machine learning algorithms (TACCO, non-negative matrix factorization, TiRank) to anchor high-resolution scRNA-seq profiles directly onto spatial transcriptomic spots. This allows inference of cell-type-specific expression within otherwise undefined tissue sub-architectures (12, 21). We propose that spatial archetype assignment must integrate three biological layers: cellular composition (identities of resident cells), topological architecture (physical proximity and spatial restriction), and functional state (signaling pathways and metabolic programs) (21, 23, 39). By synthesizing transcriptomics, spatial proteomics, routine histopathology, and regional microbiome maps, researchers can classify tumors into clinically relevant archetypal configurations (20, 30). Yet significant translational barriers remain. These include cross-platform bioinformatic incompatibilities, sampling biases inherent to small tissue microarray (TMA) cores, and a lack of standardized, regulatory-grade reporting matrices for spatial endpoints (14, 15). The key spatial profiling technologies and analytical frameworks discussed across this section are systematically summarized in **Table 1**.

**Table 1:** Major spatial profiling technologies and analytical frameworks for studying immune architecture in gastrointestinal tumors.

Technology/approach	Representative platforms or tools	Spatial resolution/data characteristics	Main strengths	Main limitations	Typical application in this review	References
Single-cell reference atlases	scRNA-seq; scATAC-seq	Single-cell molecular resolution, but native spatial coordinates are lost after tissue dissociation	Accurately defines cell states and molecular identities	Cannot preserve cell-to-cell neighborhood structure *in situ*	Serves as a reference dictionary for spatial annotation, including SPP1+/TREM2+ macrophages, FAP+ fibroblasts, and CXCL13+ CD8+ T cells	(13, 43, 80)
Array-based spatial transcriptomics	10x Genomics Visium	Broad transcriptome coverage, but each capture spot may contain multiple cells	Suitable for mapping large-scale tissue architecture and regional organization	Limited single-cell precision at cellular interfaces	Identifies immune-excluded stromal rims, angiogenic margins, lymphoid hotspots, and necrotic cores	(12, 25, 33)
High-resolution spatial transcriptomics	MERFISH; Slide-seq	Single-cell or subcellular localization with stronger emphasis on cell proximity	Well suited for studying direct cellular crosstalk and epithelial-immune interfaces	Often sacrifices full transcriptome breadth for higher spatial precision	Resolves direct apposition between malignant epithelial cells and intraepithelial lymphocytes or other immune partners	(3, 35, 81)
Multiplex spatial proteomics	mIF; IMC; MIBI; CODEX	*In situ* protein-level profiling on intact tissue sections, including FFPE samples	Validates transcriptomic predictions and detects functional proteins or post-translational states	Marker panels are preselected, and cross-platform consistency can be variable	Confirms ARG1+/TREM2+ myeloid populations, TLS organization, and invasive-front cellular neighborhoods	(15, 30, 71)
Multimodal integration and archetype scoring	TACCO; non-negative matrix factorization; TiRank	Integrates transcriptomic, proteomic, histologic, and microbiome-associated layers	Enables archetype assignment by combining cellular composition, topology, and functional state	Affected by algorithmic heterogeneity, sampling bias, and limited reporting standardization	Builds spatial archetype scoring systems for translational stratification	(14, 32, 38)

## 3 Biological building blocks of spatial organization

Before delineating the macroscopic immune archetypes, it is essential to define the fundamental, spatially regulated cellular programs that ultimately assemble to construct these functional microenvironmental niches.

### 3.1 Spatially restricted T-cell functional states

T lymphocytes in the GI tumor microenvironment show functional plasticity. That plasticity links directly to their spatial localization. Take primed effector T cells: when they actively intermingle within malignant epithelial nests, this indicates an inflamed phenotype. Such inflammation relies on intact *CXCL9/CXCL10* chemokine gradients—tissue-penetrating ones—for sustained recruitment (15, 17). Meanwhile, specialized tissue-resident memory T cells (TRM) position themselves at the tumor invasive margin or adjacent to tertiary lymphoid structures (TLS). There, they maintain durable, long-term localized immunosurveillance, independent of systemic circulation (40–42). Now consider CD8+ T cells sequestered within a dense, desmoplastic peritumoral stroma. They experience spatial restriction—denied physical access to malignant targets. These excluded cells frequently upregulate inhibitory exhaustion markers: PD-1, TIM-3, and LAG-3. Current evidence suggests this dysfunctional state is exacerbated by chronic, unresolved antigen exposure (mediated by stromal cross-presentation) and by adverse metabolic conditions within the rigid extracellular matrix (10, 15, 19).

### 3.2 Spatially localized myeloid immunosuppressive programs

The myeloid compartment includes tumor-associated macrophages (TAMs), monocytes, and myeloid-derived suppressor cells (MDSCs). Together, these cells shape the local metabolic and inflammatory milieu. High-resolution spatial mapping has revealed phenotypically distinct macrophage populations occupying non-overlapping niches (7, 43). Some subsets—IL4I1+ macrophages, for example—show antitumoral phagocytic capacity in areas of high cellular turnover. In contrast, potent immunosuppressive programs preferentially enrich in hypoxic and necrotic tumor zones. What drives these programs? Key markers: TREM2, SPP1, and ARG1 (26, 43). Within those spatially localized suppressive hubs, myeloid cells deploy multiple mechanisms to abrogate immune surveillance. They deplete local amino acids (L-arginine). They secrete immunosuppressive cytokines—IL-10 and TGF-β. They express high surface levels of inhibitory ligands, including PD-L1 and VSIG4. We argue that these synchronized activities create metabolically hostile microdomains. The consequence: impaired T-cell viability and adaptive immune evasion (2, 7, 26).

### 3.3 Stromal and vascular barriers

The gastrointestinal tumor stroma is not just a passive scaffold. It actively regulates local immune architecture. Key players here are cancer-associated fibroblasts (CAFs)—especially the FAP+ and α-SMA+ subpopulations. These cells construct a dense extracellular matrix (ECM) with extensively crosslinked collagen fibers. The result: mechanical restriction of cytotoxic lymphocyte infiltration into the tumor parenchyma (10, 27, 33). But CAFs do more than physically exclude immune cells. They actively misdirect immune responses. Through TGF-β secretion and aberrant chemokine production (notably CCL2), they preferentially recruit STAT3-activated macrophages and promote regulatory T cell (Treg) differentiation. At the same time, they repel or sequester CD8+ effector populations. This bidirectional signaling constitutes active immune misdirection rather than passive exclusion (36, 44, 45). Now consider the vasculature. Hypoxia-driven abnormal angiogenesis generates a dysfunctional, tortuous vascular network. This aberrant vasculature impedes T cell extravasation. It also exacerbates localized tissue hypoxia. We posit that a detrimental feed-forward loop then emerges: vascular dysfunction reinforces the immunosuppressive myeloid niche, which further entrenches physical barriers to effective immune infiltration (8, 24, 46).

### 3.4 Lymphoid organization and local antigen presentation

Effective antitumoral immunity depends on organized, localized antigen presentation within the TME. Diffuse immune infiltrates—even when abundant—are not enough. What matters is spatial assembly: B cells, plasma cells, and conventional dendritic cells (specifically the cross-priming cDC1 and cDC2 subsets) forming highly structured tertiary lymphoid structures (TLS) (40, 47). These TLS mark *in situ* immunocompetence. Unlike disorganized lymphocyte infiltration, these architectural aggregates replicate lymph node functions directly inside the tumor bed. They serve as protected micro-niches for active tumor antigen cross-priming, B-cell clonal expansion, immunoglobulin class switching, and generation of durable immunological memory (6, 40, 41). Current evidence indicates that TLS architectural maturity and spatial density correlate with favorable prognosis and enhanced immunotherapy response across multiple GI cancer cohorts. We argue this is not merely a correlation—it reflects a functional requirement (41, 42, 47).

## 4 Four proposed spatial immune archetypes

**Figure 1:**
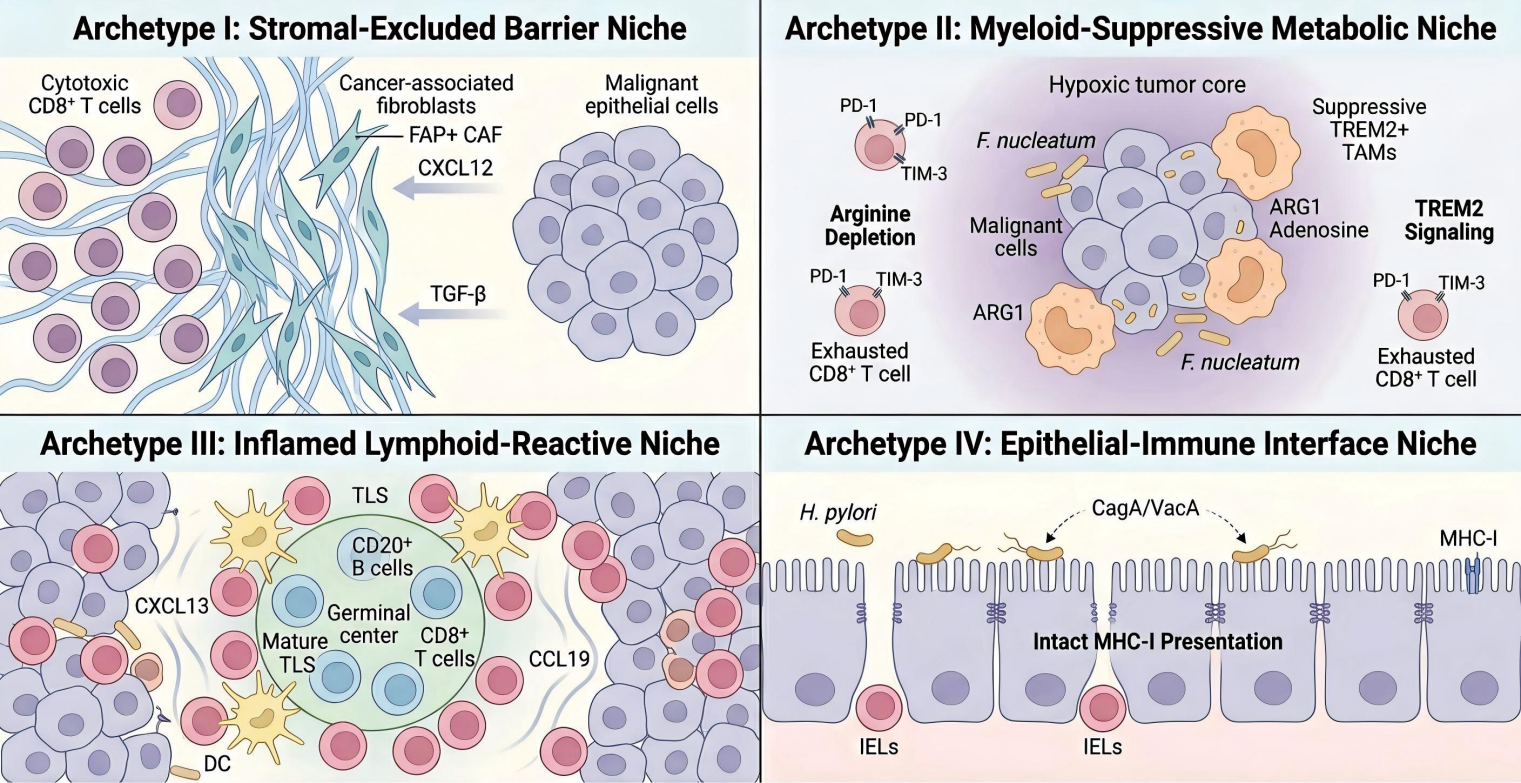
Four spatial immune archetypes in gastric and colorectal cancer. Archetype I: Stromal-Excluded Barrier Niche. Dense FAP+ CAF-enriched matrix sequesters CD8+ T cells at the invasive margin, driven by TGF-β and CXCL12/CCL2 gradients. Archetype II: Myeloid-Suppressive Metabolic Niche. SPP1+ and TREM2+ macrophages co-localize with hypoxic zones; metabolic competition disables CD8+ T cells. *F. nucleatum* amplifies this ecology. Archetype III: Inflamed Lymphoid-Reactive Niche. Mature TLS with B cell follicles and DCs support *in situ* antigen presentation. Sustained by CXCL13/CCL19/CCL21 gradients. Predominates in EBV+ and MSI-H tumors. Archetype IV: Epithelial-Immune Interface Niche. Intact epithelium with interspersed intraepithelial lymphocytes maintains immunosurveillance. MHC-I presentation governs this equilibrium, lost during immunoediting.

Through integration of the aforementioned biological building blocks and multi-omic data sets, gastrointestinal tumors potentially stratify into four distinct, functionally significant spatial immune archetypes based on our synthesis of current high-dimensional datasets (**Figure 1**). It is crucial to emphasize that these proposed archetypes are illustrative functional patterns rather than mutually exclusive diagnostic categories. In clinical reality, the TME represents a continuum where overlapping features frequently coexist.

### 4.1 Archetype I: stromal-excluded barrier niche

In this archetype, reactive effector immune cells reach the tumor periphery. But they stop there. They accumulate at the invasive margin or become sequestered within the dense stromal rim. Penetration into malignant epithelial nests fails. Spatially, this niche features extensive desmoplasia, dense collagen deposition, branched networks of activated CAFs, and vascular dysfunction. Together, these elements enforce physical compartmentalization of CD8+ T cells (10, 11, 18). The key biochemical driver is TGF-β. It acts pleiotropically: activating fibroblasts, upregulating matrix metalloproteinases, and directly suppressing T-cell effector transcription. Meanwhile, CAFs secrete high levels of CXCL12 and CCL2. These aberrant chemokine gradients misdirect and trap T cells within the stroma—reinforcing the physical barrier with a biochemical one (26, 36, 37, 48). Clinically, this archetype is enriched in diffuse-type and stroma-rich gastric cancers. Prognosis is consistently poor. In colorectal cancer (CRC), it aligns predominantly with chromosomal instability (CIN) and MSS phenotypes, corresponding to the highly fibrotic consensus molecular subtype 4 (CMS4) (20, 36, 49).

Why does PD-1/PD-L1 monotherapy typically fail here? Because T cells are spatially excluded. Preclinical and early clinical data collectively support prioritizing stromal normalization before systemic checkpoint blockade in this archetype. Current evidence points to several strategies: dual inhibition of TGF-β and PD-L1; focal adhesion kinase (FAK) inhibitors to alleviate ECM mechanical tension; CAF-depleting agents (e.g., novel FAP-targeted immunotherapies) (28, 32, 50). However, it must be acknowledged that FAP-targeted therapies currently face substantial translational challenges in clinical trials, including dose-limiting toxicities and limited single-agent efficacy. Optimizing the therapeutic index of these agents to selectively target tumor-promoting CAFs without damaging healthy FAP^+^ mesenchymal tissues remains a critical hurdle (20, 26, 51). Also, vascular reprogramming—using moderate-dose anti-angiogenic agents—can normalize endothelial barriers, facilitating T-cell extravasation and parenchymal entry before systemic immunotherapy (44, 46, 52, 53).

### 4.2 Archetype II: myeloid-suppressive metabolic niche

In this archetype, malignant cells co-localize with suppressive myeloid populations—tumor-associated macrophages (TAMs), myeloid-derived suppressor cells (MDSCs), and specialized monocytes. Together, they form spatial hubs that biochemically disable T cells at the source. The microarchitecture is consistent: SPP1+ and TREM2+ macrophages physically opposed to invading tumor glands, plus hypoxic zones, necrotic areas, deeply exhausted intralesional T cells, and intense local metabolic competition (7, 26, 43). The molecular drivers? TREM2 and SPP1. TREM2 acts as a metabolic sensor, maintaining macrophage survival in deep hypoxia via PI3K-AKT and mTOR signaling, which facilitates pathological lipid uptake. Meanwhile, high ARG1 expression depletes L-arginine—arresting T-cell proliferation and function. The suppressive milieu is further reinforced by the CD47-SIRPα axis, adenosine accumulation (CD39/CD73 pathway), and chronic NF-κB inflammatory signaling (2, 7, 45).

Now consider *Fusobacterium nucleatum* in colorectal cancer. It amplifies immunosuppression through two mechanisms. First, surface adhesin Fap2 activates the inhibitory receptor TIGIT; CbpF engages CEACAM1 via a ‘Velcro-like’ interaction, suppressing NK and T cell functions (54–56). Second, structural components and metabolites operate through distinct pathways. LPS and OMVs—not succinate—skew myeloid cells toward a tolerogenic state via TLR4-NF-κB. SCFAs bind FFAR2 to promote a Th17 phenotype. Crucially, microbiota-derived succinate impairs the cGAS-STING-IFN-β axis, downregulating Th1 chemokines (CCL5 and CXCL10). This metabolic blockade restricts CD8+ T cell migration, leading to spatial immune exclusion and intrinsic resistance to checkpoint inhibitors (5, 54, 56).

Clinically, this archetype appears in non-inflamed MSS colorectal cancers and advanced intestinal-type gastric cancers. Both show robust M2-skewed macrophage infiltration. We argue that eradicating this niche requires targeted myeloid checkpoint blockade and metabolic reprogramming. Current evidence points to several strategies: direct targeting of TREM2 or SPP1 axes; CD47/SIRPα antagonists to restore phagocytosis; metabolic interventions against arginase or adenosine signaling; PI3Kγ inhibitors for macrophage reprogramming. Also, precision microbiome-informed therapies—targeted bacteriophages or customized narrow-spectrum antibiotics—may dismantle the symbiotic *F. nucleatum* niches that drive this archetype (7, 54, 57).

### 4.3 Archetype III: inflamed lymphoid-reactive niche

This is the “immune-hot” phenotype. Spatially organized immune activation persists within intratumoral hotspots and structurally mature tertiary lymphoid structures (TLS). What does the landscape look like? Widespread T-cell infiltration deeply intermingled with malignant epithelial cells. Also precise spatial coordination among dendritic cells, B cells, and T cells. A key feature: organized lymphoid aggregates. They range from simple clusters to mature secondary follicle-like structures with proliferating germinal centers. Those centers support cyclical immune priming and effector phases (18, 22, 40). At the molecular level, type I and type II interferon (IFN) transcriptional programs drive this archetype. So does competent MHC class I and II antigen presentation machinery. And steep gradients of tissue-penetrating chemokines—CXCL13, CCL19, CCL21—recruit circulating naive and memory lymphocytes into the tumor bed (5, 55, 58).

Clinically, this configuration appears in Epstein-Barr virus (EBV)-positive and MSI-H gastric cancers. In colorectal cancer, it aligns with the high mutational burden of MSI-H/dMMR. Now note: these tumors show sensitivity to single-agent immune checkpoint inhibitors (5, 59, 60). But they frequently upregulate multiple compensatory checkpoints—PD-L1, CTLA-4, TIGIT—in response to chronic interferon-gamma (IFN-γ) exposure. In this context, amplifying and sustaining pre-existing inflammation—rather than initiating *de novo* immune responses—emerges as the more tractable therapeutic objective (57, 61, 62). We submit that rational therapeutic strategies here include dual checkpoint blockade (e.g., anti-PD-1 plus anti-CTLA-4), TLS-inducing vaccines, cytokine-based intratumoral priming (engineered IL-2 or IL-12), and agonist antibodies against costimulatory pathways such as OX40 and ICOS (40, 41, 58).

### 4.4 Archetype IV: epithelial-immune interface niche(a proposed model of early surveillance)

This archetype represents a proposed early and delicate equilibrium of active immunosurveillance. In this configuration, malignant or premalignant epithelial cells maintain direct physical contact with specialized intraepithelial lymphocytes (IELs) before the establishment of complex immune-evasive architectures (30, 57, 63). The result: active immunosurveillance. Spatially, this niche sits at the immediate margin of the epithelial barrier. Tight juxtaposition and synapse-like connections define it—not dense desmoplastic stromal bands (63, 64). At the molecular level, intact MHC class I processing and presentation tightly govern this niche. Other requirements: rapid epithelial chemokine signaling (CXCL8, CXCL10), maintenance of tight junction integrity (e.g., CLDN1), early innate PRR engagement (TLR and NOD-like receptor signaling), and contextualized mucosal crosstalk with the local microbiome (1, 5).

Now consider *Helicobacter pylori* in gastric cancer. It is the principal modifier of this interface. Through virulence factors CagA and VacA, *H. pylori* perturbs junctional integrity and dysregulates epithelial-immune sensing. It drives release of inflammatory cytokines—IL-1β, IL-6, TNF-α—while paradoxically dampening T-cell proliferation and dendritic cell maturation (56, 65, 66). Current evidence indicates that resolution or persistence of this interface dictates the outcome: effective immunosurveillance (pathogen cleared) versus permanent loss, leading to chronic atrophic gastritis and then malignancy. We acknowledge that Archetype IV is primarily observed in early mucosal lesions. While it may not persist as a dominant subtype in advanced disease, we propose its inclusion as a critical spatiotemporal baseline. It represents the surveillance state just before nascent tumors undergo widespread immunoediting and transition into the more stable, resistant configurations defined by Archetypes I, II, or III (30, 57, 61).

What threatens this transient state? Progressive loss of epithelial visibility and downregulation of surface antigens. We argue that therapeutic interventions must focus on restoring inherent antigenicity. Current strategies include epigenetic resensitization—DNA methyltransferase (DNMT) or histone deacetylase (HDAC) inhibitors—to restore MHC-I expression (67, 68). Also, bispecific T-cell engagers (BiTEs) can bridge the gap between effector T cells and concealed tumor antigens (69). Localized, low-dose radiation therapy can amplify neoantigen shedding and promote epitope spreading, fortifying the interface before irreversible evasion occurs (70).

### 4.5 Archetype overlap, plasticity, and therapy-driven transitions

These four proposed spatial immune archetypes are not mutually exclusive bins. For instance, a tumor exhibiting a stromal-excluded margin (Archetype I) may simultaneously harbor a deeply hypoxic, myeloid-suppressive core (Archetype II) within the same lesion. Nor are they static. They behave as dynamic, plastic ecological states. High-resolution spatial mapping has shown that distinct regions within a single advanced tumor can simultaneously harbor divergent archetypes, underscoring significant intratumoral spatial heterogeneity (20, 29, 71). Now consider tumor evolution from a pathological perspective. Tumors start at the epithelial-immune interface—accessible and actively surveilled. Then selective immunologic and metabolic pressures drive a transition. The destination: resistant, stromal-excluded, or myeloid-suppressive configurations as the disease advances. This evolution appears clearly in the Correa cascade (gastric cancer) and the adenoma-carcinoma sequence (colorectal cancer) (21, 28, 29). What about therapy? The goal of rationally designed combination immunotherapies is to reprogram the microenvironment. We argue that the aim is to revert tumors—from excluded or suppressive phenotypes back to an inflamed, lymphoid-reactive state. Recognizing this plasticity elevates the spatial archetype framework. It moves from a descriptive taxonomy toward a hypothetical model that may help predict and potentially modulate longitudinal disease evolution (7, 15, 57). The defining spatial features and corresponding therapeutic implications of all four archetypes are consolidated in **Table 2**.

**Table 2:** Core features and therapeutic implications of the four spatial immune archetypes in gastric and colorectal cancer.

Spatial immune archetype	Core spatial features	Key cells/molecular axes	Representative disease context	Mechanism of resistance or sensitivity to immunotherapy	Potential therapeutic strategies	References
Archetype I: Stromal-Excluded Barrier Niche	CD8+ T cells accumulate at the invasive margin or within dense stromal bands and fail to penetrate malignant epithelial nests	FAP+/alpha-SMA+ CAFs, TGF-beta, CXCL12, CCL2, abnormal vasculature	Enriched in diffuse-type and stroma-rich gastric cancers; in CRC, associated with CIN, MSS status, and CMS4	Resistance is driven mainly by physical exclusion, stromal sequestration, and chemokine misdirection, which limit the efficacy of PD-1/PD-L1 monotherapy	TGF-beta blockade, FAK inhibition, CAF-targeted approaches, vascular normalization, then combination immunotherapy	(11, 19, 44)
Archetype II: Myeloid-Suppressive Metabolic Niche	Suppressive myeloid cells co-localize with tumor glands, hypoxic areas, and necrotic regions to form metabolically hostile hubs	TREM2, SPP1, ARG1, CD47/SIRPalpha, CD39/CD73, NF-kappaB; microbiome-related signals including *Fusobacterium nucleatum*	Seen in non-inflamed MSS colorectal cancer and advanced intestinal-type gastric cancer with M2-skewed macrophage infiltration	Resistance arises from amino acid depletion, adenosine accumulation, myeloid checkpoint signaling, and microbe-associated suppression of T-cell migration and function	Targeting TREM2 or SPP1, CD47/SIRPalpha blockade, anti-adenosine or anti-arginase approaches, PI3Kgamma inhibition, and microbiome-directed therapy	(7, 26, 54, 82)
Archetype III: Inflamed Lymphoid-Reactive Niche	Dense intratumoral T-cell infiltration with mature TLS and coordinated dendritic cell-B cell-T cell organization	CXCL13, CCL19, CCL21, type I/II IFN programs, MHC-I/II, PD-L1, CTLA-4, TIGIT	Found in EBV-positive and MSI-H gastric cancer and in MSI-H/dMMR colorectal cancer	Relative sensitivity to checkpoint blockade reflects pre-existing immune activation, although compensatory checkpoint upregulation may limit durability	Dual checkpoint blockade, TLS-inducing vaccines, cytokine-based priming such as IL-2 or IL-12, and OX40/ICOS agonists	(40, 42, 58)
Archetype IV: Epithelial-Immune Interface Niche	Epithelial cells and intraepithelial lymphocytes remain in close contact at an early surveillance interface	MHC-I, CXCL8, CXCL10, CLDN1, TLR/NOD-like receptor signaling, *Helicobacter pylori*-related modulation	Most relevant in early gastric mucosal lesions, chronic gastritis-associated states, and early dysplastic colorectal lesions	Immune escape develops when epithelial antigen visibility declines and the surveillance interface is progressively lost during immunoediting	DNMT or HDAC inhibition to restore antigen presentation, BiTEs, and low-dose local radiotherapy to enhance neoantigen release and epitope spreading	(66, 83, 84)

## 5 Tissue-specific determinants of archetype prevalence in GC and CRC

### 5.1 Shared cellular origins, divergent anatomical constraints

Gastric and colorectal mucosae share embryonic endoderm origin. They use similar cellular building blocks—macrophages, CAFs, T cells, dendritic cells—to build their tumor microenvironments. But the stomach and colon have different physiological jobs. Those differences impose distinct biomechanical, chemical, and ecological constraints (1, 64). The result: tissue-specific constraints dictate how these immune programs assemble, interact, and function *in situ*.

### 5.2 Anatomy, mucus, and microbial ecology

**Figure 2:**
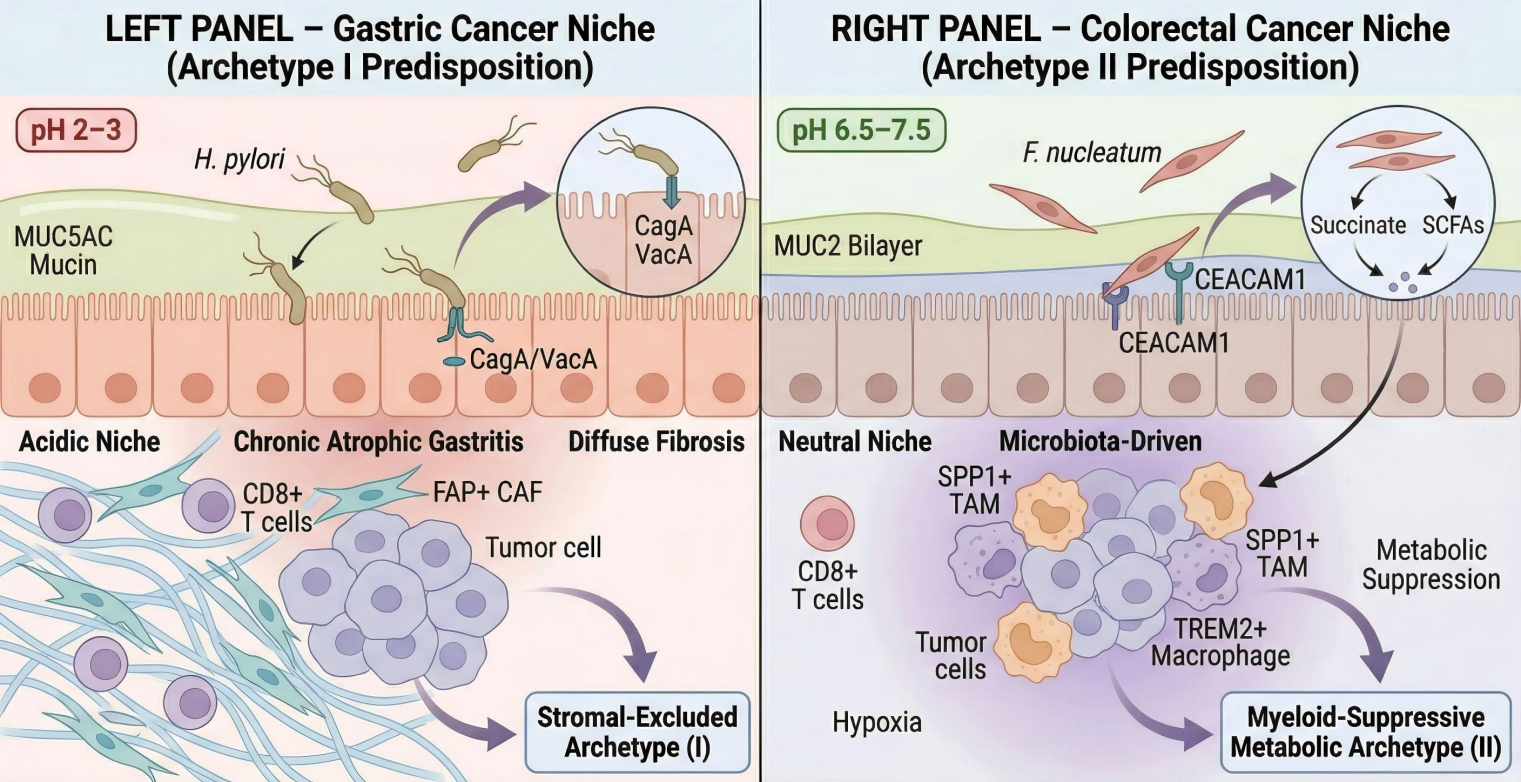
Tissue-specific constraints shape spatial immune archetypes. Left: Gastric mucosa. Low luminal pH and a continuous MUC5AC mucus layer restrict microbial diversity, favoring persistent *H. pylori* colonization. CagA and VacA virulence factors drive chronic inflammation, atrophic gastritis, and progressive stromal desmoplasia. This environment promotes the stromal-excluded Archetype I, characterized by FAP+ CAF-enriched collagen barriers and peripheral CD8+ T cell sequestration. Right: Colorectal mucosa. Neutral pH and a stratified MUC2 bilayer support dense commensal microbiota. Pathobionts such as *F. nucleatum* breach this barrier and bind CEACAM1 on epithelial cells. Bacterial metabolites (succinate, SCFAs) foster TREM2+ and SPP1+ macrophage accumulation and metabolic suppression. This ecology predisposes to the myeloid-suppressive Archetype II, wherein CD8+ T cells are disabled by local arginine depletion and adenosine signaling.

Gastric and colonic environments differ in anatomy and biochemistry. Those differences drive early niche formation. They also determine which spatial immune archetype wins out. Take the stomach. Its epithelium sits under a thick, continuous mucus layer—mostly MUC5AC mucin—adapted to withstand acidic pH and enzymatic degradation. That harsh, acidic milieu restricts microbial diversity (54, 63). The result: acid-tolerant pathogens like *H. pylori* gain control over local immune tone. This persistent infection creates localized inflammatory foci through a well-defined molecular cascade: *H. pylori* virulence factors CagA and VacA activate NF-κB and STAT3 signaling within gastric epithelial cells, triggering sustained secretion of IL-1β, IL-6, and TNF-α. These inflammatory mediators recruit FAP^+^ cancer-associated fibroblasts and stimulate TGF-β production, which drives progressive ECM remodeling with extensively crosslinked collagen networks. Over decades of this chronic fibro-inflammatory cycle, the accumulating desmoplastic stroma physically sequesters CD8^+^ T cells at the tumor periphery and actively misdirects them via CXCL12 and CCL2 gradients—hallmarks of the stromal-excluded Archetype I. It is precisely this *H. pylori*-initiated, TGF-β–sustained fibrotic cascade that mechanistically predisposes gastric tumors, particularly the genomically stable (GS) and diffuse-type subtypes, to Archetype I dominance rather than myeloid-suppressive or inflamed configurations. Now consider the colon. Its mucosa has a stratified, MUC2-dominant mucus bilayer. The lumen is neutral pH, densely packed with a diverse commensal microbiota—trillions of organisms. The colon must keep inflammation in check against a massive load of benign bacterial antigens. So its baseline immune tone is tolerogenic (1, 72). When pathogenic microbiota like *F. nucleatum* breach the MUC2 barrier, this tolerogenic baseline becomes a vulnerability. *F. nucleatum* surface adhesin Fap2 engages CEACAM1 on colonic epithelial cells and activates the inhibitory receptor TIGIT on NK and T cells, directly blunting cytolytic activity (54–56). Concurrently, bacterial LPS and outer membrane vesicles (OMVs) engage TLR4/NF-κB on myeloid precursors, skewing them toward a tolerogenic TREM2+SPP1+macrophage phenotype (26, 54, 72). Microbiota-derived succinate further impairs the cGAS–STING–IFN-β axis, downregulating Th1 chemokinesCCL5 and CXCL10 and restricting CD8^+^ T cell migration into the tumor bed (5). Within these TREM2+SPP1+ macrophage hubs, high ARG1 expression then depletes local L-arginine, arresting T cell proliferation and function at the metabolic level. This stepwise cascade—from MUC2 barrier breach, to TLR4/NF-κB–driven myeloid polarization, to ARG1-mediated metabolic T cell arrest—constitutes the molecular logic by which the colonic microenvironment is selectively conditioned toward Archetype II (myeloid-suppressive metabolic niche) dominance (2, 7, 57). The stark contrast between gastric and colonic mucosal ecologies, summarized in **Figure 2**, illustrates how tissue-specific anatomical and microbial constraints predispose tumors to divergent spatial immune archetypes.

### 5.3 Molecular subtype–archetype coupling and clinical implications

The divergent molecular cascades initiated by *H. pylori* in the stomach and *F. nucleatum* in the colon do not operate in isolation—they converge with intrinsic tumor genomics to reinforce archetype identity at the molecular subtype level. Anatomy and ecology set the stage. Then specific spatial architectures couple to molecular subtypes. The result: predictable patterns of spatial organization across GI malignancies. Take gastric cancer. EBV-positive and MSI-H subtypes favor inflamed lymphoid-reactive niches. Why? High neoantigen loads and localized viral immunogenicity drive local CXCL13 gradients (14, 47, 59). Now consider the genomically stable (GS) subtype. It shows a bias toward the stromal-excluded archetype—mirroring the desmoplasia seen in diffuse-type gastric cancers (3, 36). What about colorectal cancer? Tumors driven by chromosomal instability (CIN) and classified as CMS4 are predisposed to dense stromal exclusion. Key features: elevated TGF-β signaling and FAP+ CAF infiltration (20, 44, 49). Mapping the spatial consequences of these molecular subtypes provides a more functionally informative framework than isolated genomic cataloging, with direct implications for treatment stratification.

## 6 Clinical translation

### 6.1 Next-generation spatial biomarkers beyond bulk assays

Turning descriptive spatial biology into something clinically useful requires rigorous development and validation of spatial biomarkers. Traditional bulk assays—global tumor mutational burden (TMB), the PD-L1 Combined Positive Score (CPS)—cannot account for spatial segregation of effector cells. The result? Significant predictive inaccuracies, especially in MSS/pMMR cohorts (5, 15). Next-generation spatial indices try to fix that. They go beyond simple cell abundance. Instead, they mathematically quantify biophysical relationships within the TME. Which metrics are under clinical investigation? The absolute Euclidean distance between functional CD8+ T cells and malignant epithelial cells. Also the proximity index of suppressive TAMs to hypoxic or necrotic tumor zones (17, 46, 73). Another key metric: TLS maturity. Quantifying it allows segregation of non-functional lymphoid aggregates from fully mature, antigen-presenting secondary follicles. Advanced algorithms using multiplexed platforms (MIBI or CODEX) can now derive “stromal barrier scores.” These scores predict immune exclusion before therapy starts (7, 30, 40, 58). But clinical adoption requires more. These spatial metrics must be interpretable across institutions, analytically reproducible, and compatible with standardized, cost-effective assays using widely available pathology infrastructure (15, 18).

### 6.2 Archetype-based treatment stratification

**Figure 3:**
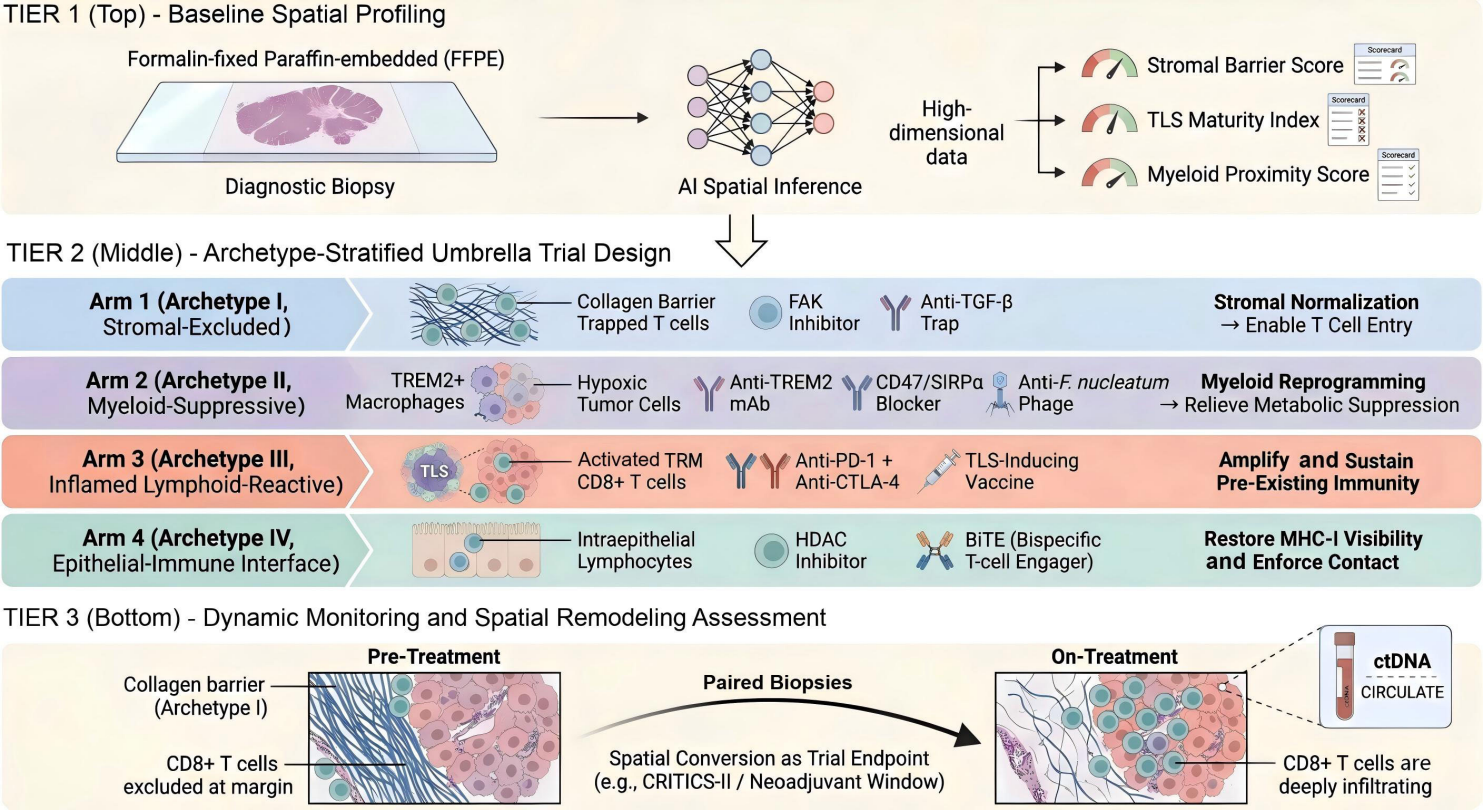
A proposed hypothetical roadmap for spatial archetype−based precision immunotherapy research. (Top) Baseline spatial profiling. Diagnostic FFPE biopsy sections undergo AI−assisted inference to derive spatial biomarkers, including stromal barrier score, TLS maturity index, and myeloid proximity score. (Middle) Archetype−stratified umbrella trial design. Patients are allocated to four therapeutic arms based on their dominant spatial archetype. Archetype I (stromal−excluded): stromal normalization via FAK inhibition or *TGF−β* trap. Archetype II (myeloid−suppressive): myeloid reprogramming with anti−TREM2, CD47/SIRPα blockade, or microbiota−directed phage therapy. Archetype III (inflamed lymphoid−reactive): amplification of pre−existing immunity using dual checkpoint blockade or TLS−inducing vaccines. Archetype IV (epithelial−immune interface): restoration of antigen visibility via HDAC inhibition or bispecific T−cell engagers. (Bottom) Dynamic monitoring. Paired pre− and on−treatment biopsies enable assessment of spatial conversion (e.g., Archetype I → Archetype III) as a trial endpoint, exemplified by neoadjuvant window−of−opportunity studies such as CRITICS−II.

Overcoming primary resistance requires a shift. Precision oncology should move beyond isolated genomic mutations or broad drug classes. Instead, treatment stratification could potentially be informed by the proposed spatial archetypes of the TME. A hypothetical clinical roadmap for future validation, from baseline spatial profiling to stratified therapy and dynamic monitoring, is outlined in **Figure 3** (18, 57). Take stromal-excluded tumors (Archetype I). Here, the initial priority is dismantling physical and biochemical stromal barriers. That means deploying TGF-β–sequestering antibodies, FAK inhibitors to relieve ECM mechanical tension, and vascular-normalizing agents—all before standard PD-1 blockade (44, 46, 52, 53). For myeloid-suppressive metabolic tumors (Archetype II), the goal is to disrupt local suppressive hubs. Current evidence points to several options: monoclonal antibodies targeting TREM2 or SPP1 signaling, CD47/SIRPα blockade to restore phagocytosis, and small molecules that block adenosine metabolism in hypoxic zones. We also note that microbiota-directed interventions—for example, targeted bacteriophages against *F. nucleatum*—can help alleviate myeloid-driven suppression in colorectal cancer (5, 7, 26, 54).

What about inflamed lymphoid-reactive tumors (Archetype III)? Baseline immune activation is already vigorous yet constrained by overlapping compensatory checkpoints. The clinical task shifts to amplifying response and ensuring durability. Dual checkpoint inhibition (ipilimumab plus nivolumab) fits here, along with TLS-inducing vaccines and next-generation cytokines that sustain the intratumoral cytokine milieu (40, 58, 61). Finally, for nascent tumors with loss of the epithelial-immune interface (Archetype IV), the primary objective is rapid resensitization of malignant epithelium. Options include epigenetic modifiers to restore MHC-I presentation, or bypassing natural antigen presentation entirely—using bispecific T-cell engagers (BiTEs) and engineered cellular therapies that forcibly reinstate direct immune-tumor interactions (8, 61).

The clinical failures reviewed in Section 6.3 underscore a critical lesson: rational target selection alone is insufficient without prospective spatial stratification of the patient population. The near-zero response rate of bintrafusp alfa in CMS4 MSS colorectal cancer most likely reflects enrollment of a molecularly heterogeneous patient cohort, rather than true failure of the TGF-β/PD-L1 dual-blockade concept itself. Spatial profiling of pre-treatment biopsies could have identified, and excluded, patients whose dominant resistance mechanism was myeloid-suppressive (Archetype II) or epithelial-immune loss (Archetype IV), rather than stromal exclusion (Archetype I)—the very archetype bintrafusp alfa was designed to dismantle (20, 57). Similarly, the redundant immunosuppressive pathways that rescued TREM2+ macrophage niches after TREM2 inhibitor monotherapy suggest that Archetype II patients require multi-axis combination strategies, not single-target depletion (7, 26). These observations collectively argue that spatial archetype identity—not genomic subtype alone—should serve as the primary stratification criterion for future trial enrollment, with stromal barrier scores, TREM2+/SPP1+ proximity indices, and TLS maturity scores serving as co-primary eligibility biomarkers alongside CMS classification (20, 40, 73).

### 6.3 Longitudinal spatial profiling for on-treatment monitoring

A baseline diagnostic biopsy offers only a static snapshot. The tumor microenvironment is anything but static—it is hostile and constantly changing. Advanced spatial profiling changes this picture. It allows longitudinal monitoring of architectural shifts while patients remain on treatment. How? By systematically comparing spatially mapped pre-treatment and on-treatment biopsies. Clinicians can then ask: is the tumor’s architecture remodeling in the intended direction (18, 30)? Consider an example. Spatial analyses can empirically confirm whether a novel stromal-targeting agent has converted an immune-excluded Archetype I lesion into a highly reactive Archetype III profile. That is precisely where neoadjuvant and window-of-opportunity trials—such as the CRITICS-II study in resectable gastric cancer—prove invaluable. These settings provide direct, empirical evaluation of spatial conversion. Investigators can quantify post-treatment T-cell expansion immediately adjacent to residual tumor beds. That yields critical proof-of-concept for therapeutic spatial remodeling (74, 75).

Now translate archetype-guided therapies into practice. We need a sober look at recent trials. Take bintrafusp alfa—an anti-PD-L1/TGF-β trap fusion protein. It was hypothesized to dismantle the desmoplastic matrix in CMS4 MSS colorectal cancers. Yet in the BINTRA study and advanced CRC cohorts, the objective response rate stood at only 3.1%. Worse, in completely resected MSS mCRC patients with ctDNA positivity, the ctDNA clearance rate was 0% (76, 77). The trial ended early. What about targeting the myeloid niche? Trials combining TREM2 inhibitors with PD-1 blockade failed to meet efficacy thresholds, even when macrophages were depleted. Redundant immunosuppressive pathways appear to take over (7). Current evidence points to a way forward through complementary monitoring strategies rather than competing paradigms. Liquid biopsy approaches—such as the ctDNA-based CIRCULATE-CRC study—operate at the systemic level, dynamically tracking circulating tumor DNA, MRD, and the emergence of resistant clones across the entire tumor burden (5, 20, 78). *In situ* spatial profiling, by contrast, resolves the local microenvironmental architecture: it can confirm whether a stromal-targeting agent has genuinely remodeled the ECM, whether TREM2+ macrophage hubs have been dismantled, or whether TLS maturity has increased within the treated lesion (7, 18, 40). These two modalities address fundamentally different biological questions and should therefore be positioned as complementary tools within a unified monitoring framework—not as alternatives to one another. Integrating systemic ctDNA kinetics with longitudinal spatial archetype mapping provides the most complete picture of treatment response: one captures what is happening in the bloodstream, the other captures what is happening inside the tumor (5, 7, 20). But true spatial translation depends on neoadjuvant trials. The CRITICS-II phase II study in gastric cancer is one example. It provides ‘window-of-opportunity’ specimens to validate spatial algorithms like the TRACERx-PHLEX stromal barrier score. The goal: reliably link architecture to pathological responses. These efforts represent a critical translational step toward converting spatial immune archetype concepts into actionable clinical decisions (18, 74, 75, 78).

### 6.4 Archetype-stratified umbrella trial design

Leveraging these spatial insights offers a conceptual framework for reimagining clinical trial design. We suggest a move toward exploring archetype-enriched, biomarker-stratified umbrella designs in research settings. Under this framework, patients with traditionally refractory MSS/pMMR tumors would no longer be enrolled based on isolated genetic mutations. Instead, prospective stratification would rely on their baseline, high-dimensional spatial architecture (5, 7, 57). Take two examples. A patient with a myeloid-suppressive niche (Archetype II) goes into a TREM2 inhibitor or microbiome-modulator arm. Another patient showing a stromal-excluded profile (Archetype I) would be allocated to a TGF-β blockade cohort (7, 20, 49). Critically, this spatial stratification should be enforced as a mandatory eligibility criterion—not merely a stratification factor—so that patients whose tumors show mixed or transitional archetype profiles (e.g., co-existing stromal and myeloid suppression, as visualized by intratumoral spatial heterogeneity mapping) are allocated to combination arms targeting both resistance axes simultaneously. This design principle directly addresses the redundancy failures observed in prior single-target trials and provides the mechanistic rationale for evaluating sequential or concurrent stromal-plus-myeloid reprogramming strategies in future phase II basket studies (7, 20, 32).

What will it take to make this work? Three things, in our view. First, widespread establishment of harmonized, cross-institutional digital pathology pipelines. Second, adoption of shared, open-source computational reporting standards. Third, rigorous validation of spatial endpoints across large-scale, prospective multicenter cohorts. Only then can we ensure universal clinical applicability—and could potentially pave the way for validating spatial archetype stratification as a future companion diagnostic standard, enabling the kind of archetype-matched trial enrollment that current genomic classifiers alone cannot provide (18, 20, 40).

## 7 Challenges and future directions

### 7.1 Technical and analytical barriers to clinical translation

Spatial biology has advanced our understanding of tumor biology, but its translation from discovery research to routine clinical practice faces major logistical and analytical hurdles. High-plex assays, especially sequencing-based spatial transcriptomics, remain cost-prohibitive for routine diagnostic use. They also cannot meet standard oncology turnaround times. Clinical specimens present additional challenges. Archival formalin-fixed, paraffin-embedded (FFPE) samples, the most widely available clinical material, show highly variable fixation quality and extensive antigen degradation. These issues directly impair RNA integrity and reduce proteomic yield (15, 33, 49). Current commercial platforms share another limitation: they rely almost exclusively on thin, two-dimensional tissue sections. This approach cannot provide true volumetric resolution. Critical, highly localized interaction hubs—located just microns deeper in the tissue block—may therefore go undetected (23, 35, 40).

Cross-platform discordance between sequencing and imaging modalities remains a major issue. Computational reproducibility across different bioinformatic algorithms also hinders biomarker standardization (14, 15, 18). Specifically, algorithms used to infer cell-cell communication from single-cell or spatial transcriptomic data have inherent limitations. These tools generate hypotheses about putative ligand-receptor interactions based on expression correlations. But they produce high false-positive rates, as transcriptomic inference is indirect. These predictions lack direct validation at the protein level—where post-translational modifications, subcellular localization, and actual binding affinities dictate functional interactions. They also assume static interaction states. As a result, they cannot fully capture the dynamic, context-dependent nature of cellular communication within the TME (14, 30, 79). We posit that the field must exercise rigorous caution. While the individual cellular components described here (e.g., FAP+ CAFs, TREM2+ macrophages) are well-supported by literature, their organization into four discrete, recurrent archetypes remains a conceptual synthesis. Mapping spatial associations and identifying close cellular proximity does not definitively establish direct mechanistic causality. In silico findings are hypothesis-generating only. They require orthogonal validation through functional assays, spatial proteomics, and *in vivo* or ex vivo models prior to clinical translation (18, 32, 39). By integrating disparate biological phenomena into a cohesive taxonomy, we provide a structured scaffold for interpreting complex spatial datasets, though the definitive boundaries of these archetypes await further experimental confirmation (18, 79).

### 7.2 Future directions: AI integration and 4D spatiotemporal mapping

The next major advances in spatial oncology will come primarily from computational progress, not just hardware innovations. AI-assisted digital pathology and sophisticated deep learning models are rapidly advancing toward routine analysis of standard hematoxylin and eosin (H&E) stained slides. These multimodal neural networks can accurately infer complex, high-dimensional spatial transcriptomic archetypes directly from basic morphological features. This will drastically reduce both assay costs and clinical turnaround times (18, 20, 49). Large-scale cross-platform benchmarking initiatives are needed to standardize archetype scoring and mitigate persistent bioinformatic discordance (14, 18, 30). The most important upcoming development is longitudinal four-dimensional (4D) mapping, which explicitly incorporates the temporal vector. This will enable investigators to trace the precise spatiotemporal trajectories of individual tumor and immune cells as they dynamically respond to therapeutic pressure. This approach will move spatial biology beyond static, descriptive taxonomies. It will culminate in the development of predictive, clinically actionable classification systems (15, 18, 30).

## 8 Conclusion

Immune checkpoint inhibitors show limited clinical efficacy in MSS/pMMR gastric and colorectal cancers. This cannot be fully explained by cellular absence, intrinsic anergy, or isolated genetic mutations. Instead, it is shaped by the restrictive, intricate spatial organization of the tumor microenvironment. Current high-dimensional multi-omic evidence indicates that antitumoral immunity in gastrointestinal malignancies appears to be organized into highly structured, functionally relevant spatial archetypes—offering an alternative perspective to stochastic cellular abundance. We have systematically delineated four distinct architectural states: the stromal-excluded barrier, the myeloid-suppressive metabolic hub, the inflamed lymphoid-reactive hotspot, and the delicate epithelial-immune interface. This comprehensive spatial framework links foundational spatial biology to the development of actionable spatial biomarkers and the rational design of precision combinatorial therapies. For the large cohort of patients with traditionally refractory GI cancers, targeted disruption and active remodeling of these spatial niches is biologically rational. Shifting the therapeutic focus from isolated cellular targets to systematic reprogramming of the tumor’s spatial ecology may extend the durable survival benefits of modern immunotherapy to more patients. Furthermore, while current spatial multi-omics platforms excel at mapping topological architecture, many of the cell-cell interactions—such as putative ligand-receptor pairs—and functional states inferred computationally from spatial transcriptomics remain correlative. These in silico predictions have not yet been systematically validated. Therefore, establishing high-throughput functional validation pipelines utilizing *in vitro* patient-derived organoids (PDOs) and *in vivo* humanized animal models must be prioritized as a critical future research direction to confirm the mechanistic dependency of these spatial archetypes.

## Glossary

BiTE: bispecific T-cell engager
CAF: cancer-associated fibroblast
cDC1: conventional dendritic cell 1
CIN: chromosomal instability
CODEX: co-detection by indexing
CRC: colorectal cancer
CTLA-4: cytotoxic T-lymphocyte-associated protein 4
DNMT: DNA methyltransferase
ECM: extracellular matrix
FFPE: formalin-fixed, paraffin-embedded
GS: genomically stable
HDAC: histone deacetylase
IELs: intraepithelial lymphocytes
IFN-gamma: interferon-gamma
L-arginine: L-arginine
MDSC: myeloid-derived suppressor cell
MIBI: multiplexed ion beam imaging
MRD: minimal residual disease
MSS: microsatellite stable
ORR: objective response rate
PD-L1: programmed death-ligand 1
PRR: pattern recognition receptor
SCFAs: short-chain fatty acids
TAM: tumor-associated macrophage
TIL: tumor-infiltrating lymphocyte
TMA: tissue microarray
TME: tumor microenvironment
TRM: tissue-resident memory T cell
BiTEs: bispecific T-cell engagers
CAFs: cancer-associated fibroblasts
cDC2: conventional dendritic cell 2
CMS4: consensus molecular subtype 4
CPS: Combined Positive Score
ctDNA: circulating tumor DNA
dMMR: deficient mismatch repair
EBV: Epstein-Barr virus
FAK: focal adhesion kinase
GC: gastric cancer
H&E: hematoxylin and eosin
IEL: intraepithelial lymphocyte
IFN: interferon
IMC: imaging mass cytometry
LPS: lipopolysaccharide
MDSCs: myeloid-derived suppressor cells
mIF: multiplex immunofluorescence
MSI-H: microsatellite instability-high
OMVs: outer membrane vesicles
PD-1: programmed cell death protein 1
pMMR: proficient mismatch repair
scATAC-seq: single-cell assay for transposase-accessible chromatin sequencing
scRNA-seq: single-cell RNA sequencing
TAMs: tumor-associated macrophages
TLS: tertiary lymphoid structure
TMB: tumor mutational burden
Treg: regulatory T cell
PDOs: patient-derived organoids.

## References

[1] Al-Ishaq RK Koklesova L Kubatka P Büsselberg D . Immunomodulation by gut microbiome on gastrointestinal cancers: focusing on colorectal cancer. Cancers. (2022) 14:2140. doi: 10.3390/cancers14092140 35565269 PMC9101278

[2] Hu S Heng H Yang F Wang M Liu G Xiang Y . The metabolism-immune axis in colorectal cancer: remodeling the tumor microenvironment through metabolite signaling. Front Immunol. (2025) 16:1735873. doi: 10.3389/fimmu.2025.1735873 41488637 PMC12757272

[3] Yan H Liu Y . Advances in spatial multi-omics in gastric cancer. Cells. (2026) 15:535–64. doi: 10.3390/cells15060535 41892326 PMC13025482

[4] Colombo A Cordio S Gebbia V Piazza D Porretto CM . Immunotherapy in pMMR/MSS metastatic colorectal cancer. Nat Cell Sci. (2024) 2:17–22. doi: 10.61474/ncs.2023.00031

[5] Cataldi C Karaoğlan BB Liotta E De Dosso S . Decoding immunotherapy response in colorectal cancer: translational insights beyond MSI. Cancers. (2026) 18:852–71. doi: 10.3390/cancers18050852 41827785 PMC12984286

[6] Dai N-N Hu M-Y Wang J-P Dai Z-H Xu L Ye T-W . Tertiary lymphoid structures in the microenvironment of colorectal cancer: exploring new therapeutic targets. Cancer Immunol Immunother. (2025) 74:245–58. doi: 10.1007/s00262-025-04108-x 40542829 PMC12182536

[7] Dai Y Luo X Zhang L Yan J . Roles of immunosuppressive myeloid states in colorectal cancer checkpoint inhibitor non-response: single-cell and spatial proteomics, and reprogramming approaches. Front Immunol. (2026) 16:1742654. doi: 10.3389/fimmu.2025.1742654 41567197 PMC12816290

[8] Zhang Z Wu Y . Research progress on mechanisms of tumor immune microenvironment and gastrointestinal resistance to immunotherapy: mini review. Front Immunol. (2025) 16:1641518. doi: 10.3389/fimmu.2025.1641518 40787471 PMC12331752

[9] Yan S Wang W Feng Z Xue J Liang W Wu X . Immune checkpoint inhibitors in colorectal cancer: limitation and challenges. Front Immunol. (2024) 15:1403533. doi: 10.3389/fimmu.2024.1403533 38919624 PMC11196401

[10] Mao C Zhang M Zhou K Hong Y Han Y Zhao L . Integrative single-cell, spatial, and bulk transcriptomics reveal an FMR1–FTO axis linked to the immune-excluded phenotype in gastric cancer. Front Immunol. (2026) 17:1713267. doi: 10.3389/fimmu.2026.1713267 41878447 PMC13006672

[11] Lin Z Wang J Ma Y Zhu Y Li Y Xiao Z . Cancer-associated fibroblasts establish spatially distinct prognostic niches in subcutaneous colorectal cancer mouse model. Cancers. (2025) 17:2402. doi: 10.3390/cancers17142402 40723284 PMC12293927

[12] Jin Y Zuo Y Li G Liu W Pan Y Fan T . Advances in spatial transcriptomics and its applications in cancer research. Mol Cancer. (2024) 23:129–53. doi: 10.1186/s12943-024-02040-9 38902727 PMC11188176

[13] Ren L Huang D Liu H Ning L Cai P Yu X . Applications of single-cell omics and spatial transcriptomics technologies in gastric cancer (review). Oncol Lett. (2024) 27:152–71. doi: 10.3892/ol.2024.14285 38406595 PMC10885005

[14] Liu Y Gao F Cheng Y Qi L Yu H . Applications and advances of multi-omics technologies in gastrointestinal tumors. Front Med. (2025) 12:1630788. doi: 10.3389/fmed.2025.1630788 40771479 PMC12325369

[15] Liu F Li G Zheng Y Liu Y Liu K . Multiplex imaging analysis of the tumor immune microenvironment for guiding precision immunotherapy. Front Immunol. (2025) 16:1617906. doi: 10.3389/fimmu.2025.1617906 40718485 PMC12289683

[16] Tsujikawa T Mitsuda J Ogi H Miyagawa-Hayashino A Konishi E Itoh K . Prognostic significance of spatial immune profiles in human solid cancers. Cancer Sci. (2020) 111:3426–34. doi: 10.1111/cas.14591 32726495 PMC7540978

[17] Chen Y Jia K Sun Y Zhang C Li Y Zhang L . Predicting response to immunotherapy in gastric cancer via multi-dimensional analyses of the tumour immune microenvironment. Nat Commun. (2022) 13:4851. doi: 10.1038/s41467-022-32570-z 35982052 PMC9388563

[18] Zhong L Li Q Xiong T Lin S Wang K Li G . Spatial omics in gastrointestinal oncology: recent advances, therapeutic insights, and clinical translation. J Cancer. (2026) 17:515–23. doi: 10.7150/jca.127381 41869445 PMC13003551

[19] Kim KT Lee MH Shin S-J Cho I Kuk JC Yun J . Decorin as a key marker of desmoplastic cancer-associated fibroblasts mediating first-line immune checkpoint blockade resistance in metastatic gastric cancer. Gastric Cancer. (2025) 28:12–26. doi: 10.1007/s10120-024-01567-6 39520589

[20] Jung S . From CMS to iCMS/IMF: developing roadmap to precision therapy in colorectal cancer. Int J Mol Sci. (2025) 26:11086. doi: 10.3390/ijms262211086 41303568 PMC12652317

[21] Avraham-Davidi I Mages S Klughammer J Moriel N Imada S Hofree M . Spatially defined multicellular functional units in colorectal cancer revealed from single cell and spatial transcriptomics. eLife. (2025) 14:RP104815. doi: 10.7554/eLife.104815 41384492 PMC12700527

[22] Pelka K Hofree M Chen JH Sarkizova S Pirl JD Jorgji V . Spatially organized multicellular immune hubs in human colorectal cancer. Cell. (2021) 184:4734–52.e20. doi: 10.1016/j.cell.2021.08.003 34450029 PMC8772395

[23] Mo C-K Liu J Chen S Storrs E Targino Da Costa ALN Houston A . Tumour evolution and microenvironment interactions in 2D and 3D space. Nature. (2024) 634:1178–86. doi: 10.1038/s41586-024-08087-4 39478210 PMC11525187

[24] Xiao Y Li Y Zhao H . Spatiotemporal metabolomic approaches to the cancer-immunity panorama: a methodological perspective. Mol Cancer. (2024) 23:202–34. doi: 10.1186/s12943-024-02113-9 39294747 PMC11409752

[25] Liang W Zhu Z Xu D Wang P Guo F Xiao H . The burgeoning spatial multi-omics in human gastrointestinal cancers. PeerJ. (2024) 12:e17860. doi: 10.7717/peerj.17860 39285924 PMC11404479

[26] Wang J Wang Y Liu Y Yang R . SPP1+ macrophages in tumor immunosuppression: mechanisms and therapeutic implications. Front Immunol. (2025) 16:1711015. doi: 10.3389/fimmu.2025.1711015 41425545 PMC12714887

[27] Sun Q Li S Lou J Wang X Xu X . Recent advances in tumour microenvironment impact immunotherapy resistance in gastric cancer. Crit Rev Oncol Hematol. (2025) 215:104837. doi: 10.1016/j.critrevonc.2025.104837 40618861

[28] Zhang G Zhang X Pan W Chen X Wan L Liu C . Dissecting the spatial and single-cell transcriptomic architecture of cancer stem cell niche driving tumor progression in gastric cancer. Adv Sci. (2025) 12:2413019. doi: 10.1002/advs.202413019 39950944 PMC12079437

[29] Ma H Srivastava S Ho SWT Xu C Lian BSX Ong X . Spatially resolved tumor ecosystems and cell states in gastric adenocarcinoma progression and evolution. Cancer Discov. (2025) 15:767–92. doi: 10.1158/2159-8290.CD-24-0605 39774838 PMC11962405

[30] Di Mauro F Arbore G . Spatial dissection of the immune landscape of solid tumors to advance precision medicine. Cancer Immunol Res. (2024) 12:800–13. doi: 10.1158/2326-6066.CIR-23-0699 38657223 PMC11217735

[31] Xiao J Yu X Meng F Zhang Y Zhou W Ren Y . Integrating spatial and single-cell transcriptomics reveals tumor heterogeneity and intercellular networks in colorectal cancer. Cell Death Dis. (2024) 15:326–37. doi: 10.1038/s41419-024-06598-6 38729966 PMC11087651

[32] Wang Q Ni Y Lu S Zhang B Ji J Cai Q . Multi-dimensional omics integrated machine learning framework identifies macrophage-fibroblast-tumor co-infiltration patterns to predict prognosis in gastric cancer. NPJ Digital Med. (2025) 9:10–28. doi: 10.1038/s41746-025-02179-9 41360923 PMC12775079

[33] Huang B Chen Y Yuan S . Application of spatial transcriptomics in digestive system tumors. Biomolecules. (2024) 15:21–39. doi: 10.3390/biom15010021 39858416 PMC11761220

[34] Danan CH Katada K Parham LR Hamilton KE . Spatial transcriptomics add a new dimension to our understanding of the gut. Am J Physiol Gastrointest Liver Physiol. (2023) 324:G91–8. doi: 10.1152/ajpgi.00191.2022 36472345 PMC9870576

[35] Du Y Ding X Ye Y . The spatial multi-omics revolution in cancer therapy: precision redefined. Cell Rep Med. (2024) 5:101740. doi: 10.1016/j.xcrm.2024.101740 39293393 PMC11525011

[36] Lee SH Lee D Choi J Oh HJ Ham I-H Ryu D . Spatial dissection of tumour microenvironments in gastric cancers reveals the immunosuppressive crosstalk between CCL2+ fibroblasts and STAT3 -activated macrophages. Gut. (2025) 74:714–27. doi: 10.1136/gutjnl-2024-332901 39580151 PMC12013559

[37] Sabbadini F Bertolini M De Matteis S Mangiameli D Contarelli S Pietrobono S . The multifaceted role of TGF-β in gastrointestinal tumors. Cancers. (2021) 13:3960. doi: 10.3390/cancers13163960 34439114 PMC8391793

[38] Che G Yin J Wang W Luo Y Chen Y Yu X . Circumventing drug resistance in gastric cancer: a spatial multi-omics exploration of chemo and immuno-therapeutic response dynamics. Drug Resist Update. (2024) 74:101080. doi: 10.1016/j.drup.2024.101080 38579635

[39] Yang S Gu C Miao X Zuo H Xu W Zhang Y . Single-cell and spatial transcriptome profiling identifies the immunosuppressive spatial niche in KRAS -mutant colorectal cancer. J Immunother Cancer. (2025) 13:e013763. doi: 10.1136/jitc-2025-013763 41475845 PMC12766835

[40] Deng S Chen Y Song B Wang H Huang S Wu K . Tertiary lymphoid structures in cancer: spatiotemporal heterogeneity, immune orchestration, and translational opportunities​​. J Hematol Oncol. (2025) 18:97–126. doi: 10.1186/s13045-025-01754-7 41219991 PMC12606831

[41] Zhao L Jin S Wang S Zhang Z Wang X Chen Z . Tertiary lymphoid structures in diseases: immune mechanisms and therapeutic advances. Sig Transduct Target Ther. (2024) 9:225–68. doi: 10.1038/s41392-024-01947-5 39198425 PMC11358547

[42] Xie Y Peng H Hu Y Jia K Yuan J Liu D . Immune microenvironment spatial landscapes of tertiary lymphoid structures in gastric cancer. BMC Med. (2025) 23:59–77. doi: 10.1186/s12916-025-03889-3 39901202 PMC11792408

[43] Chu X Zhang Y Cheng S China CL Beijing 102206 . Heterogeneity of tumor-infiltrating myeloid cells in era of single-cell genomics. Chin J Cancer Res. (2022) 34:543–53. doi: 10.21147/j.issn.1000-9604.2022.06.01 36714348 PMC9829493

[44] Henriques A Salvany-Celades M Nieto P Palomo-Ponce S Sevillano M Hernando-Momblona X . TGF-β builds a dual immune barrier in colorectal cancer by impairing T cell recruitment and instructing immunosuppressive SPP1+ macrophages. Nat Genet. (2025) 57:3050–65. doi: 10.1038/s41588-025-02380-2 41203813

[45] Chen B Tang H Zheng X Xie F Yu P Lyu Y . Spatial and functional dissection of cancer-associated fibroblasts-mediated immune modulation in H. pylori-associated gastric cancer. Mol Cancer. (2025) 24:282–302. doi: 10.1186/s12943-025-02490-9 41194113 PMC12590883

[46] Lim SH An M Heo YJ Lee H Min B-H Mehta A . Distinct spatially resolved tumor microenvironment trajectories define benefit from ramucirumab plus pembrolizumab in refractory PD-L1+ gastric cancer. Cancer Immunol Res. (2026) 14:307–17. doi: 10.1158/2326-6066.CIR-25-0625 41252599 PMC12767546

[47] Wang Y Zhang G Zhang X Liu G Zhang L Chen L . Single-cell and spatial transcriptomics implicate a prognostic function of tertiary lymphoid structures in gastric cancer. Nat Commun. (2025) 16:10435. doi: 10.1038/s41467-025-65421-8 41290589 PMC12647882

[48] Mo S Wang Y Xiong R Ma L Xu M Wang L . Spatially defined danger zone shapes gastric cancer progression through CCDC80+ fibroblast–induced CD8+ T cell dysfunction. Apoptosis. (2026) 31:91–113. doi: 10.1007/s10495-026-02287-1 41793512 PMC12967633

[49] Kantha A Das D Pai E Kumar T Pandey M . Consensus molecular subtypes (CMS) classification: a progress towards subtype-driven treatments in colorectal cancer. World J Surg Oncol. (2025) 24:1–19. doi: 10.1186/s12957-025-04117-1 41276825 PMC12763931

[50] Zhu B Zheng C Xu H Zheng Y Liu Y Li P . Mass spectrometry-based multi-omics analysis elucidates immune microenvironmental characteristics and the risk of distant metastasis in N1c colorectal cancer. Front Immunol. (2026) 17:1590042. doi: 10.3389/fimmu.2026.1590042 41782869 PMC12953441

[51] Wu T Li X Zheng F Liu H Yu Y . Intercellular communication between FAP+ fibroblasts and SPP1+ macrophages in prostate cancer via multi-omics. Front Immunol. (2025) 16:1560998. doi: 10.3389/fimmu.2025.1560998 40438108 PMC12116517

[52] Stanley KA Holmen SL . Targeting FAK to potentiate immune checkpoint therapy in solid tumors. J Cancer Immunol. (2025) 7:99–108. doi: 10.33696/cancerimmunol.7.109 41019359 PMC12462869

[53] Qian C Liu C Liu W Zhou R Zhao L . Targeting vascular normalization: a promising strategy to improve immune–vascular crosstalk in cancer immunotherapy. Front Immunol. (2023) 14:1291530. doi: 10.3389/fimmu.2023.1291530 38193080 PMC10773740

[54] Sorino J Della Mura M Ingravallo G Cazzato G Pizzimenti C Zuccalà V . Fusobacterium nucleatum and gastric cancer: an emerging connection. Int J Mol Sci. (2025) 26:7915. doi: 10.3390/ijms26167915 40869236 PMC12386183

[55] Roelands J Kuppen PJK Ahmed EI Mall R Masoodi T Singh P . An integrated tumor, immune and microbiome atlas of colon cancer. Nat Med. (2023) 29:1273–86. doi: 10.1038/s41591-023-02324-5 37202560 PMC10202816

[56] Gusmaulemova A Kurentay B Bayanbek D Kulmambetova G . Comparative insights into fusobacterium nucleatum and helicobacter pylori in human cancers. Front Microbiol. (2025) 16:1677795. doi: 10.3389/fmicb.2025.1677795 41195396 PMC12583222

[57] Anderson KG Braun DA Buqué A Gitto SB Guerriero JL Horton B . Leveraging immune resistance archetypes in solid cancer to inform next-generation anticancer therapies. J Immunother Cancer. (2023) 11:e006533. doi: 10.1136/jitc-2022-006533 37399356 PMC10314654

[58] Baxevanis CN Sofopoulos M Tsitsilonis OE Gritzapis AD . Exploring the pivotal functions of tertiary lymphoid structures in cancer prognosis and immunotherapy outcomes. Cancers. (2025) 17:3754. doi: 10.3390/cancers17233754 41374956 PMC12691252

[59] Hou W Zhao Y Zhu H . Predictive biomarkers for immunotherapy in gastric cancer: current status and emerging prospects. Int J Mol Sci. (2023) 24:15321. doi: 10.3390/ijms242015321 37895000 PMC10607383

[60] Ma J Li J Hao Y Nie Y Li Z Qian M . Differentiated tumor immune microenvironment of epstein-barr virus-associated and negative gastric cancer: implication in prognosis and immunotherapy. Oncotarget. (2017) 8:67094–103. doi: 10.18632/oncotarget.17945 28978018 PMC5620158

[61] Peshin S Bashir F Kodali NA Dharia A Zaiter S Singal S . Immunotherapy in GI cancers: lessons from key trials and future clinical applications. Antibodies. (2025) 14:58–79. doi: 10.3390/antib14030058 40700298 PMC12285940

[62] Sun F Gao X Wang W Zhao X Zhang J Zhu Y . Predictive biomarkers in the era of immunotherapy for gastric cancer: current achievements and future perspectives. Front Immunol. (2025) 16:1599908. doi: 10.3389/fimmu.2025.1599908 40438098 PMC12116377

[63] Pelaseyed T Bergström JH Gustafsson JK Ermund A Birchenough GMH Schütte A . The mucus and mucins of the goblet cells and enterocytes provide the first defense line of the gastrointestinal tract and interact with the immune system. Immunol Rev. (2014) 260:8–20. doi: 10.1111/imr.12182 24942678 PMC4281373

[64] Suárez LJ Arboleda S Angelov N Arce RM . Oral versus gastrointestinal mucosal immune niches in homeostasis and allostasis. Front Immunol. (2021) 12:705206. doi: 10.3389/fimmu.2021.705206 34290715 PMC8287884

[65] González-Stegmaier R Aguila-Torres P Villarroel-Espíndola F . Historical and molecular perspectives on the presence of helicobacter pylori in latin america: a niche to improve gastric cancer risk assessment. Int J Mol Sci. (2024) 25:1761. doi: 10.3390/ijms25031761 38339039 PMC10855479

[66] Wizenty J Tacke F Sigal M . Responses of gastric epithelial stem cells and their niche to helicobacter pylori infection. Ann Transl Med. (2020) 8:568–568. doi: 10.21037/atm.2020.02.178 32775369 PMC7347775

[67] Yang Z Chu B Tu Y Li L Chen D Huang S . Dual inhibitors of DNMT and HDAC remodels the immune microenvironment of colorectal cancer and enhances the efficacy of anti-PD-L1 therapy. Pharmacol Res. (2024) 206:107271. doi: 10.1016/j.phrs.2024.107271 38906202

[68] Han R Zhou H Peng B Yu S Zhu J Chen J . Synergistic integration of HDAC inhibitors and individualized neoantigen therapy (INT): a next-generation combinatorial approach for cancer immunotherapy. Vaccines. (2025) 13:550–78. doi: 10.3390/vaccines13060550 40573881 PMC12197479

[69] Burton KA Metropulos AE Kamath SD Munshi HG Principe DR . Bispecific T-cell engager therapy for gastrointestinal cancers. Cancer Lett. (2026) 646:218417. doi: 10.1016/j.canlet.2026.218417 41806779

[70] Lussier DM Alspach E Ward JP Miceli AP Runci D White JM . Radiation-induced neoantigens broaden the immunotherapeutic window of cancers with low mutational loads. Proc Natl Acad Sci. (2021) 118:e2102611118. doi: 10.1073/pnas.2102611118 34099555 PMC8214694

[71] Scheuermann S Kristmann B Engelmann F Nuernbergk A Scheuermann D Koloseus M . Unveiling spatial complexity in solid tumor immune microenvironments through multiplexed imaging. Front Immunol. (2024) 15:1383932. doi: 10.3389/fimmu.2024.1383932 38566984 PMC10985204

[72] Zhao Q Maynard CL . Mucus, commensals, and the immune system. Gut Microbes. (2022) 14:2041342. doi: 10.1080/19490976.2022.2041342 35239459 PMC8903774

[73] Zhang S Deshpande A Verma BK Wang H Mi H Yuan L . Integration of clinical trial spatial multiomics analysis and virtual clinical trials enables immunotherapy response prediction and biomarker discovery. Cancer Res. (2024) 84:2734–48. doi: 10.1158/0008-5472.CAN-24-0943 38861365 PMC12010747

[74] Slagter AE Jansen EPM Van Laarhoven HWM Van Sandick JW Van Grieken NCT Sikorska K . CRITICS-II: a multicentre randomised phase II trial of neo-adjuvant chemotherapy followed by surgery versus neo-adjuvant chemotherapy and subsequent chemoradiotherapy followed by surgery versus neo-adjuvant chemoradiotherapy followed by surgery in resectable gastric cancer. BMC Cancer. (2018) 18:877–89. doi: 10.1186/s12885-018-4770-2 30200910 PMC6131797

[75] Verheij M Hartgrink H Polee M Beerepoot L Hoekstra R van Hillegersberg R . CRITICS-II: a multicenter randomized phase II trial of neo-adjuvant chemotherapy followed by surgery versus neo-adjuvant chemotherapy and subsequent chemoradiotherapy followed by surgery versus neo-adjuvant chemoradiotherapy followed by surgery in resectable gastric cancer. doi: 10.1200/JCO.2026.44.2_suppl.283 PMC6131797 30200910

[76] Spira A Wertheim MS Kim EJ Tan B Lenz H-J Nikolinakos P . Bintrafusp alfa: a bifunctional fusion protein targeting PD-L1 and TGF-β, in patients with pretreated colorectal cancer: results from a phase I trial. Oncologist. (2023) 28:e124–7. doi: 10.1093/oncolo/oyac254 36576431 PMC9907041

[77] Morris VK Overman MJ Lam M Parseghian CM Johnson B Dasari A . Bintrafusp alfa, an anti-PD-L1:TGFβ trap fusion protein, in patients with ctDNA-positive, liver-limited metastatic colorectal cancer. Cancer Res Commun. (2022) 2:979–86. doi: 10.1158/2767-9764.CRC-22-0194 36382087 PMC9648419

[78] Zeng Z Yang B Liao Z . Biomarkers in immunotherapy-based precision treatments of digestive system tumors. Front Oncol. (2021) 11:650481. doi: 10.3389/fonc.2021.650481 33777812 PMC7991593

[79] Gao S Qin S Wang D Wang A Zhu L Li Y . A spatially resolved atlas of gastric cancer characterises a lymphocyte-aggregated region. Nat Commun. (2026) 17:2059. doi: 10.1038/s41467-026-68612-z 41593079 PMC12948980

[80] Deng G Zhang X Chen Y Liang S Liu S Yu Z . Single-cell transcriptome sequencing reveals heterogeneity of gastric cancer: progress and prospects. Front Oncol. (2023) 13:1074268. doi: 10.3389/fonc.2023.1074268 37305583 PMC10249727

[81] Nagasawa S Zenkoh J Suzuki Y Suzuki A . Spatial omics technologies for understanding molecular status associated with cancer progression. Cancer Sci. (2024) 115:3208–17. doi: 10.1111/cas.16283 39042942 PMC11447966

[82] Xie Z Zheng G Niu L Du K Li R Dan H . SPP1 macrophages in colorectal cancer: markers of Malignancy and promising therapeutic targets. Genes Dis. (2025) 12:101340. doi: 10.1016/j.gendis.2024.101340 40092488 PMC11907465

[83] Yan T Sun H Liao Y Zhou J . Helicobacter pylori mediated niche environment aberrations promote the progression of gastric cancer. Genes Dis. (2024) 11:101207. doi: 10.1016/j.gendis.2024.101207 38882015 PMC11176646

[84] Fung C Tan S Nakajima M Skoog EC Camarillo-Guerrero LF Klein JA . High-resolution mapping reveals that microniches in the gastric glands control helicobacter pylori colonization of the stomach. PLoS Biol. (2019) 17:e3000231. doi: 10.1371/journal.pbio.3000231 31048876 PMC6497225

